# Hybrid 2-[^18^F] FDG PET/MRI in premanifest Huntington’s disease gene-expansion carriers: The significance of partial volume correction

**DOI:** 10.1371/journal.pone.0252683

**Published:** 2021-06-11

**Authors:** Marie N. N. Hellem, Tua Vinther-Jensen, Lasse Anderberg, Esben Budtz-Jørgensen, Lena E. Hjermind, Vibeke Andrée Larsen, Jørgen E. Nielsen, Ian Law

**Affiliations:** 1 The Neurogenetics Clinic, Danish Dementia Research Centre, Rigshospitalet, University of Copenhagen, Copenhagen, Denmark; 2 Department of Clinical Physiology, Nuclear Medicine and PET, Rigshospitalet, University of Copenhagen, Copenhagen, Denmark; 3 Department of Public Health, Section of Biostatistics, University of Copenhagen, Copenhagen, Denmark; 4 Department of Radiology, Rigshospitalet, University of Copenhagen, Copenhagen, Denmark; University of Minnesota Duluth, UNITED STATES

## Abstract

**Background:**

Huntington’s disease (HD) is an inherited, progressive neurodegenerative disease that has no cure. Striatal atrophy and hypometabolism has been described in HD as far as 15 years before clinical onset and therefore structural and functional imaging biomarkers are the most applied biomarker modalities which call for these to be exact; however, most studies are not considering the partial volume effect and thereby tend to overestimate metabolic reductions, which may bias imaging outcome measures of interventions.

**Objective:**

Evaluation of partial volume effects in a cohort of premanifest HD gene-expansion carriers (HDGECs).

**Methods:**

21 HDGECs and 17 controls had a hybrid 2-[^18^F]FDG PET/MRI scan performed. Volume measurements and striatal metabolism, both corrected and uncorrected for partial volume effect were correlated to an estimate of disease burden, the CAG age product scaled (CAP_S_).

**Results:**

We found significantly reduced striatal metabolism in HDGECs, but not in striatal volume. There was a negative correlation between the CAP_S_ and striatal metabolism, both corrected and uncorrected for the partial volume effect. The partial volume effect was largest in the smallest structures and increased the difference in metabolism between the HDGEC with high and low CAP_S_ scores. Statistical parametric mapping confirmed the results.

**Conclusions:**

A hybrid 2-[^18^F]FDG PET/MRI scan provides simultaneous information on structure and metabolism. Using this approach for the first time on HDGECs, we highlight the importance of partial volume effect correction in order not to underestimate the standardized uptake value and thereby the risk of overestimating the metabolic effect on the striatal structures, which potentially could bias studies determining imaging outcome measures of interventions in HDGECs and probably also symptomatic HD.

## 1. Introduction

Huntington’s disease (HD) is a progressive, autosomal dominantly inherited neurodegenerative disease caused by an expansion of a trinucleotide (CAG) repeat in the huntingtin (*HTT*) gene. The symptoms are a combination and variation of involuntary movements, behavioral changes and other psychiatric symptoms as well as cognitive impairment leading to dementia [1].

The nature of the disease makes it possible to identify premanifest HD gene-expansion carriers (HDGECs) but not to predict symptoms or age at onset. The CAG age product (CAP) score is based on current age, CAG repeat length and a constant, applied on premanifest individuals used as an index of cumulative toxicity of mutant huntingtin and to estimate time to predicted onset of motor symptoms. CAP score scaled (CAP_S_) gives an estimate of the 5 years 50–50 risk of motor onset. This calculation has a bootstrap-adjusted area under the curve value of 0.7172 which indicates a relatively strong ability to predict the risk of diagnosis [2].

Structural brain imaging is by far the most applied and well documented technique to demonstrate longitudinal structural changes in premanifest and manifest HDGECs [3]. Multiple imaging studies with computer tomography (CT), magnetic resonance imaging (MRI) and functional imaging studies using 2-[^18^F]fluoro-2-deoxy-D-glucose (2-[^18^F]FDG) positron emission tomography (PET) imaging have been carried out.

Using MRI, volume reductions in the basal ganglia, primarily in the striatum, was described and even registered as far as 15 years before motor onset [4]. More general atrophy including global loss of white and grey matter tissue outside the striatum is also described in studies on premanifest HDGECs [4,5]and the loss of white matter has been shown to precede both the loss of grey matter and onset of motor symptoms [6].

Studies on glucose metabolism in the brain by 2-[^18^F]FDG PET revealed hypometabolism in the Caudate nucleus, Putamen and right frontal lobe in premanifest HDGECs [7,8]. One study reported reduced glucose metabolism in the Caudate nucleus in premanifest HDGECs and hypothesized that this fact may improve the prediction of age at onset [9]. Hypermetabolism in the thalamus prior to symptom onset that decreases to subnormal levels on manifestation of symptoms has also been reported [10]. Reduction in striatal glucose metabolism was hypothesized to precede striatal volume reductions [11], however, this has not been confirmed.

All imaging techniques suffer, however, from the partial volume effect (PVE) in which limited scanner resolution causes the measured signal to appear reduced because more than one tissue type occupies the same voxel leading to the loss of contrast between two adjacent regions [12]. Thus, it is difficult to judge if a reduction in glucose metabolism in the basal ganglia is more pronounced than can be explained solely by atrophy. As striatal volume is reduced with increasing CAP_S_ score and symptom severity it could be important to correct for the PVE to appropriately ascertain if any physiological change in the patient group could be explained by structural volume change per se. Paradoxically, the data analysis strategies used in the majority of the literature, however, warp the brain to a standard space and reduce PET resolution through spatial filtering that will increase the PVE.

To evaluate PVE, we present, to our knowledge, an unprecedented study of partial volume corrected (PVC) 2-[^18^F]FDG using a hybrid PET/MRI scanner in premanifest HDGECs which allow a simultaneous characterization of the structural and metabolic brain changes in premanifest HDGECs. We conclude that not using PVC may lead to underestimation of the metabolism in the affected regions which potentially may bias imaging outcome measures of e.g. interventions in HDGECs.

## 2. Material and methods

### 2.1 Participants

We included 22 participants from the Neurogenetics Clinic, Danish Dementia Research Centre, Copenhagen, Denmark with a CAG repeat ≥39 and a Unified Huntington’s Disease Rating scale-99 (UHDRS-motor) total motor score <5, a Mini Mental State Examination (MMSE) score ≥24 and a Montreal Cognitive Assessment (MoCA) score ≥19. The participants were part of a larger cohort recruited from January 2012 to March 2013 and previously described in detail [13]. Demographics can be seen in **Table 1** along with exclusion criteria in supplementary material **S1 Table** in S1 File. Participant demographics was described using median and range. We registered gender, age, CAG repeat, CAP_S_ score, MoCA score and UHDRS-motor score [13]. All premanifest HDGECs had a CAPs score calculated at the entry of the study from the equation (Age x (CAG-33.66))/432.3326 [2]. As a control group we included 17 healthy participants, 14 of which were offspring from HDGECs carrying a CAG repeat of less than 30. The additional three were age and gender matched non-family controls. All participants provided written, informed consent and the study was approved by the Ethics Committee of the Capital Region of Denmark (H2-2011-085). We had to exclude one participant due to metallic prosthesis, which left us with 21 HDGECs.

**Table 1:** Demographic data.

	Controls	Premanifest HDGECs
N	17	21
Gender (M/F)	9/8	14/7
Age (years)	40 (26–57)	37 (27–51)
CAG repeat length	<30	42 (39–46)
CAP_S_ score	NR	0.71 (0.41–1.06)
MoCA	28.5	29
UHDRS-motor score	NR	1 (0–4)

All values are given in medians and range.

HDGECs: HD gene-expansion carriers.

NR: Not Relevant.

CAP_S_ score: CAG age product scores, Calculated from the equation (Age x (CAG– 33.66))/432.3326.

MoCA: Montreal Cognitive Assessment.

UHDRS-TMS: Unified Huntington’s Disease Rating Scale–Total Motor Score.

Age distribution and MoCA were not significantly different between the two groups.

### 2.2 Imaging procedures and analysis

Participants received a single bed position 10 min static PET acquisition initiated 45 minutes after injection of 200 MBq 2-[^18^F]FDG simultaneously with MRI on a hybrid PET/MRI scanner (Siemens Biograph mMR, Siemens Healthcare, Erlangen, Germany [14]).

All subjects fasted for at least 6 hours before undergoing scanning. Their blood glucose levels were measured just before injection of the radionuclide-containing solution to ensure values below 8 mmol/L.

The head was fixed in a standard 16 channel head-neck coil. Images were reconstructed with 3D Ordinary Poisson-Ordered Subset Expectation Maximization (OP-OSEM) with 4 iterations, 21 subsets, zoom 2.5, and 3.0 mm Gaussian post filtering on 344 × 344 matrices (0.8 × 0.8 × 2.0 mm^3^ voxels) in line with the clinical protocol used at our institution [15]. For all images default random, scatter, and dead time correction were applied. For attenuation correction a same-day reference low-dose CT image was acquired (120 kVp, 36 mAs, 74 slices, 0.6 × 0.6 × 3 mm^3^ voxels) of the head using a whole-body PET/CT system (Biograph TruePoint 40 and Biograph TruePoint 64, Siemens Healthcare) [16].

The MRI protocol was without contrast lasting approximately 20 min and consisted of 5 standard clinical sequences (see supplementary material).

MRI scans were read clinically by a certified neuroradiologist (VAL) blinded to clinical and genetic status of participants. Furthermore, each 2-[^18^F]FDG PET were evaluated blinded to diagnosis by subject specific voxel-based analysis comparing regional activity normalized to cerebellar activity with age matched healthy subjects on an individual basis using a standard clinical PET platform (Siemens Syngo.via, MI Neurology, Database (VE20A)). The PET scans were scored clinically (by IL) from 1 to 5 based on features of statistical surface projections or the subcortical uptake pattern relative to cortex and deviations from the control group (Supplementary material). Normal (1) with striatal uptake at or above the level of the cortex; possibly abnormal (2) with minor regional reduction in striatum below cortex with an SD < -2.0 or cortical reductions either with a SD < - 2.0 that may not signify disease; slightly abnormal (3) with striatal metabolic values below the cortex or cortical reductions either with SD values between -4.0 and -2.0; moderately abnormal (4) with striatal metabolic reduction or cortical reductions of approximately half of the cortical levels either with SD values < -4.0; strongly abnormal (5) with striatal metabolic reduction lower than half of the cortical levels or cortical reductions either with SD values < -6.0.

We compared the separate volumes and metabolism of the Caudate nucleus and Putamen in controls and HDGECs s. Based on this data we chose to analyze the striatum as a combined structure. The anatomical volumes were automatically segmented based on the T1 MPRAGE MR images in a priori selected grey matter brain structures, namely the cortex, the hippocampi, the amygdalae, the thalami, the striatum (sum of the Caudate nucleus, Putamen, Globus Pallidus), and the cerebellum using the FreeSurfer software suite version 6.0 [17]. The average 2-[^18^F]FDG activity, as a surrogate measure of glucose metabolism, was measured in all regions, and PVC using the Symmetric Geometric Transfer Matrix (SGTM) method implemented in the PETSurfer toolkit of FreeSurfer [18–20]. The SGTM was the recommended method in a recent head to head comparison of 3 commonly used techniques [21]. Volume measures and metabolic activity were expressed as average of right and left. The volumes were normalized to total intracranial volume (ICV) to account for differences in brain size [22], and the metabolic activities were normalized to the PVC cerebellar grey matter value and reported as the relative standardized uptake value (SUVR).

### 2.3 Statistics

To document the effects, if any, on performing PVC on 2-[^18^F]FDG PET images we performed linear regression analysis of the difference between average striatal 2-[^18^F]FDG PET with and without PVC as a function of absolute and uncorrected MRI volumes (mL). The MRI volumes were uncorrected, as it is absolute size that determines the PVE. Thus, the PVE should have the largest impact on 2-[^18^F]FDG PET value on the smallest MRI volume.

We used unpaired *t*-tests corrected for multiple comparisons by Bonferroni-Holm between HDGECs and controls in the comparison of the average striatal ICV corrected MRI volumes and PVC 2-[^18^F]FDG PET metabolism. In modeling the data, several combinations of response variables and covariates were used. Linear regression analysis was performed between both the average striatal ICV corrected MRI volume and striatal metabolism vs. the CAP_S_ scores, respectively. Age correction is implicit in the CAP_S_ scores. The computations were performed in R. Multiple comparison correction of p-values were done using the Bonferroni-Holm method as implemented in the base library of R [23].

The diagnostic accuracy of the average striatal ICV corrected MRI volume and the PVC 2-[^18^F]FDG PET striatal metabolism was computed using the library pROC [23] available through R yielding the optimal thresholds and the related sensibility, specificity and accuracy. P-values were considered significant when ≤0.05.

### 2.4 2-[^18^F]FDG PET statistical parametric mapping analysis

A supplementary voxel based analysis on the group level was performed to further corroborate the results and identify changes that could not be encompassed within the region based analysis above. The 2-[^18^F]FDG PET brain images were spatially normalized to the Montreal Neurologic Institute (MNI) PET template using a 12 parameter affine transformation and a nonlinear warp in statistical parametric mapping (SPM 12) software (Wellcome Trust Centre for Neuroimaging, UCL, London, UK) [24]. The images were smoothed with a 12 mm Gausian filter to account for individual variations in anatomy and increase the signal-to-noice ratio. In the statistical analyses, global activity was adjusted for age and normalized to the cerebellar activity measured by applying a standard anatomical atlas in MNI space (WFU PickAtlas, http://fmri.wfubmc.edu/software/PickAtlas) [25,26]. A grey matter mask of activity > 80% of max was used. Significant differences for correlation analysis with CAPs score were evaluated both at the voxel level and at cluster level statistics. At the voxel level using a threshold of p<0.05 corrected for multiple independent comparisons using Family-Wise-Error (FWE), and at thecluster level significance using a threshold of p<0.05 using FWE for cluster above 200 at an uncorrected threshold of p<0.01. Age was linearly adjusted as a “nuisance” covariate in the general linear model used. All statistically significant results were listed with the location designated as anatomical region, stereotactic coordinates in MNI space with corresponding peak T value and significance level.

## 3. Results

### 3.1 Visual evaluation

#### 3.1.1 MRI

In the group of controls, all had a normal structural MRI. In the group of premanifest HDGECs, one participant had a small cyst (11 x 6mm) inferior to the left Putamen. Another participant had an arachnoid cyst in the posterior fossa causing local cerebellar hypoplasia and one participant had minimal unspecific gliosis in the left hemisphere, whereas the remaining participants had unremarkable MRIs. None of the findings interfered with MRI segmentation.

#### 3.1.2 2-[^18^F]FDG PET

In the control group 14 were classified as normal, while three were regarded as slightly abnormal either because of a statistical feature in the statistical surface projections or an impression of striatal hypometabolism. Of the HDGECs, 9 were visually evaluated as normal, 4 possibly abnormal, 5 slightly abnormal and 3 were moderately abnormal and no one were strongly abnormal (**S1 Fig** in S1 File**)**.

**Fig 1:**
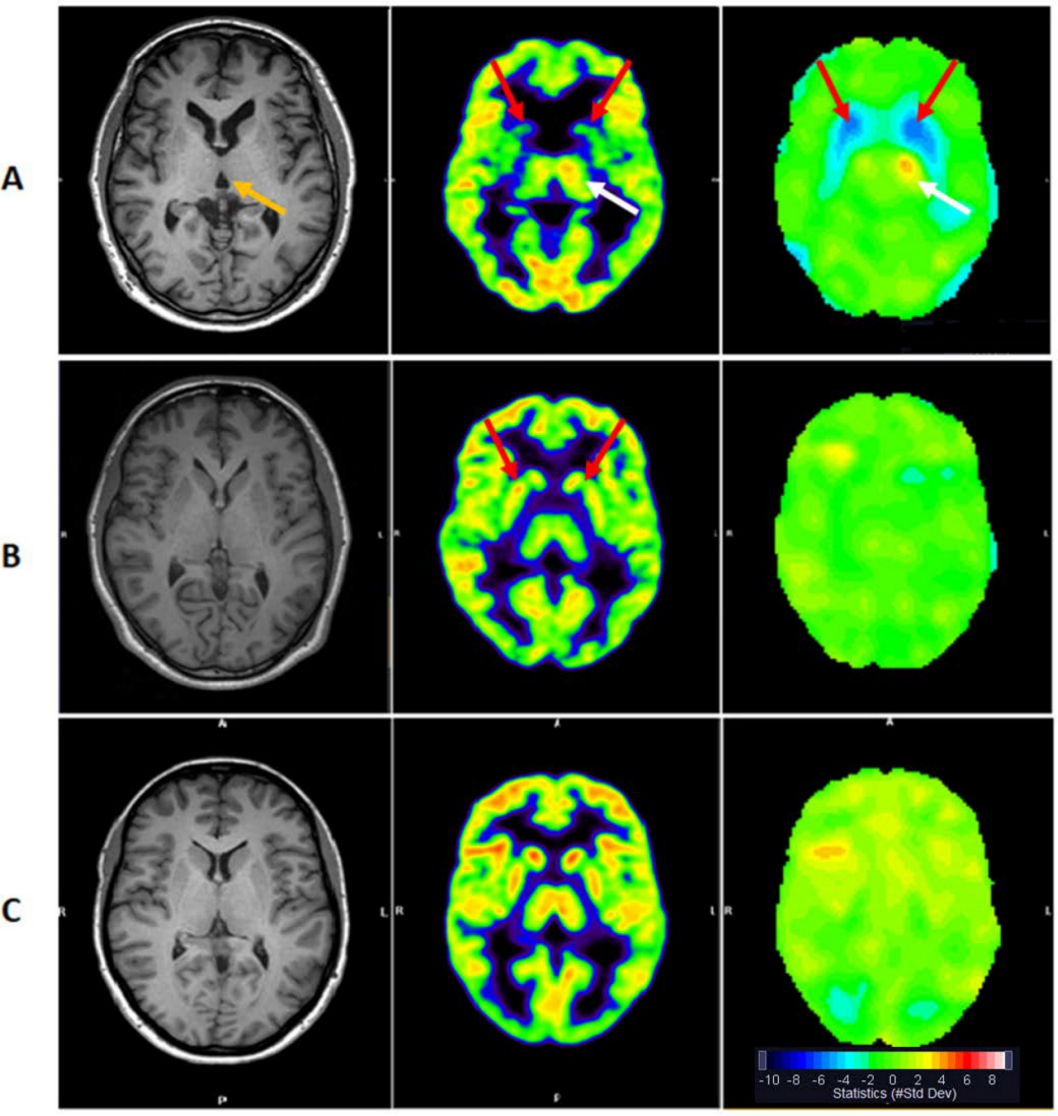
Axial T1 weighted MRI (left), 2-[^18^F]FDG PET (center) and statistical analysis (right) at the same level in standard anatomical space in two HD gene-expansion carriers (A, B), and a healthy control for comparison (C). Both patients and control had normal MRI. Patient A. 2-[^18^F]FDG PET SUVR is moderately abnormal with symmetrically decreased metabolism in the Striata (red arrows). Possibly compensatory increased metabolism in the left thalamus (white arrow). CAP_S_ score 1,04. Patient B. 2-[^18^F]FDG PET SUVR show possibly abnormal metabolism with the impression of slight striatal metabolic reduction (red arrows) compared to the cortex although within normal range in the statistical map. CAP_S_ score 0.91. The figure shows the range of abnormal 2-[^18^F]FDG PET uptake in patients with high CAP_S_ scores. Note the enlargement of the third ventricle in patient A (orange arrow), that appear as a spurious SPM correlation (**Table 5**). The statistical maps (right) compare regional activity without PVC normalized to cerebellar activity to a group of age matched controls using (MI Neurology, Siemens) displayed in Z-scores with color scale shown.

The 3 HDGECs classified as moderately abnormal on their 2-[^18^F]FDG PET uptake, all had CAP_S_ scores above 0.8 (range 0.84 to 1.04), while the group of slightly abnormal ranged from 0.68 to 1.06. In the group visually evaluated as normal the range of CAP_S_ scores was from 0.41 to 1.04 and the last group classified as possibly abnormal had CAP_S_ scores from 0.46 to 0.91 (**Fig 1)**.

### 3.2 Difference between striatal metabolism and volume in controls and HDGECs

Comparing the separate volumes of Caudate nucleus and Putamen in controls and HDGECs we found no significant difference in their MRI measured volumes while their metabolic activity in both structures were significantly lower in HDGECs. Therefore, we analyzed the striatum as a combined structure.

When we compared HDGECs to controls, we found a significantly lower (1.50 ±0.14; corrected p = 0.03) PVC average striatal metabolism (SUVR) in the HDGEC group compared to controls while the average striatal ICV corrected MRI volumes after correction for multiple comparisons was not significantly different in HDGECs (1.23±0.15 corrected p = 0.13) (**Table 2**). This is in line with our finding of non-significantly different slopes comparing the regression lines for controls and HDGECs in regard to SUVR with and without PVC plotted against MRI volume with and without correction for ICV. We did however find significantly different intercepts. So, HDGECs have significantly lower metabolism than the controls also after adjustment for MRI volume.

**Table 2:** Intracranial volume (ICV) corrected MRI volumes and Partial volume corrected (PVC) 2-[^18^F]FDG brain metabolism.

	A. ICV corrected MRI volumes			B. PVC 2-[^18^F]FDG brain metabolism			C. 2-[^18^F]FDG brain metabolism, no PVC		
ROI	Controls Mean ± SD (%)	HDGECs Mean ± SD (%)	P value corrected	Controls Mean ± SD	HDGECs Mean ± SD	P value corrected	Controls Mean ± SD (%)	HDGECs Mean ± SD	P value corrected
Hippocampus	0.55 ± 0.04	0.54 ± 0.05	1	1.04 ± 0.08	1.03 ± 0.06	1	0.92 ± 0.06	0.91 ± 0.05	1
Amygdala	0.23 ± 0.02	0.22 ± 0.02	1	0.91 ± 0.11	0.89 ± 0.08	1	0.85 ± 0.08	0.82 ± 0.04	0.38
Thalamus	1.02 ± 0.08	1.00 ± 0.06	1	1.27 ± 0.1	1.29 ± 0.09	1	1.15 ± 0.07	1.16 ± 0.07	1
Cortex	31.72 ± 2.02	31.38 ± 1.65	1	1.60 ± 0.15	1.54 ± 0.13	0.796	1.32 ± 0.11	1.30 ± 0.09	1
Striatum	1.33 ± 0.11	1.23 ± 0.15	0.13	1.63 ± 0.15	1.49 ± 0.14	0.03*	1.42 ± 0.12	1.29 ± 0.14	0.02

A. an unpaired t-test of the difference in MRI volumes between Huntington’s disease gene-expansion carriers (HDGECs) and controls, expressed in % of the intracranial volume (ICV). B. an unpaired t-test of the difference in partial volume corrected 2-[^18^F]fluoro-2-deoxy-D-glucose (2-[^18^F]FDG) metabolism relative to cerebellar cortex (SUVR) between HDGECs and controls. C. an unpaired t-test of the difference in 2-[^18^F]fluoro-2-deoxy-D-glucose (2-[^18^F]FDG) metabolism relative to cerebellar cortex (SUVR) between HDGECs and controls. The volumes are averaged between the left and right hemisphere. Significant results after correction for multiple comparisons by Bonferroni-Holm marked by *.

### 3.3 CAPs score correlated to striatal volume, glucose metabolism and the difference with and without partial volume correction

**Fig 2:**
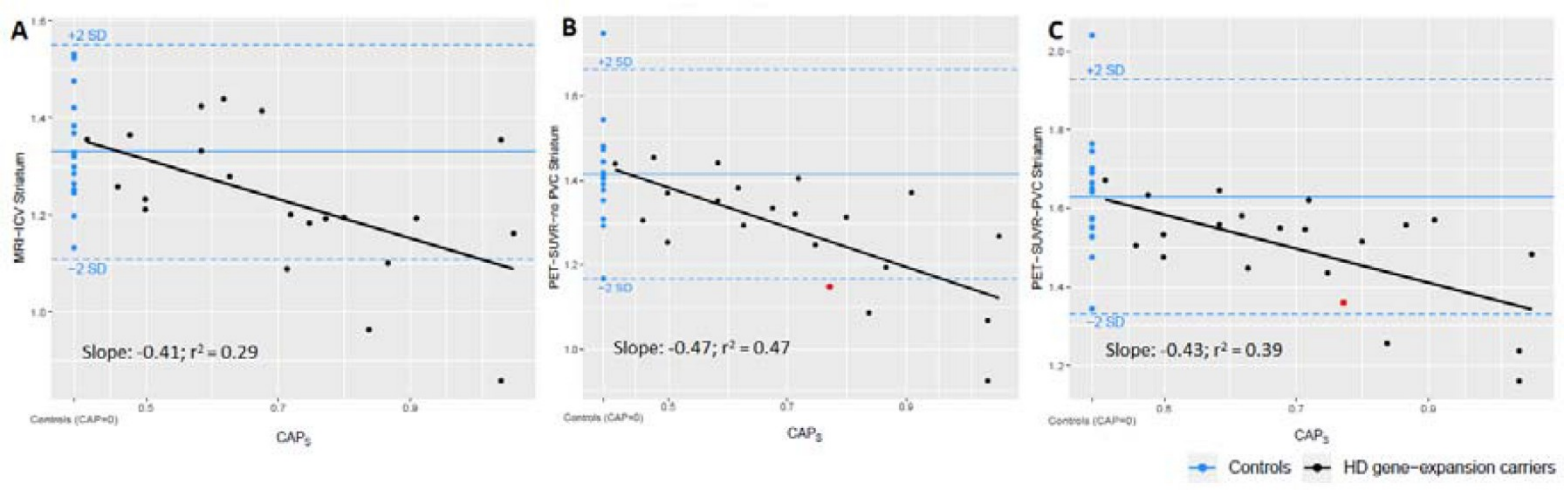
Scatter plots showing the striatal metrics derived as the average of both sides of the MRI volume (%) corrected for intra cranial volume (A), the uncorrected 2-[^18^F]FDG uptake (SUVR) normalized to cerebellar cortex (B), and the partial volume corrected 2-[^18^F]FDG uptake (SUVR) normalized to cerebellar cortex (C) vs. the CAP_S_ score. The regression lines were all significant and are shown with slope and r^2^. For comparisons controls are plotted in blue at CAP_S_ score 0 showing mean +/- 2 SD. The measurement, marked in red, represents the participant, who without PVC is under the -2 SD.

We investigated whether the striatal volume and metabolism was correlated to the CAP_S_ scores respectively and found a significant negative association between both striatal metabolism, both corrected and uncorrected for the partial volume effect, and CAP_S_ score (slope -0.43; p = 0.003; r^2^ = 0.39 and slope -0.47; p = 0.007; r^2^ = 0.0.47, respectively) and MRI Striatal volume corrected for ICV and CAP_S_ score (slope -0.41; p = 0.01; r^2^ = 0.29) (**Fig 2**). Three HDGECs were below -2 SD on the plot of metabolism corrected for partial volume and four fell below on the plot without partial volume correction. On the plot of ICV corrected MRI volume, 4 of the HDGECs were below -2SD.

**Fig 3:**
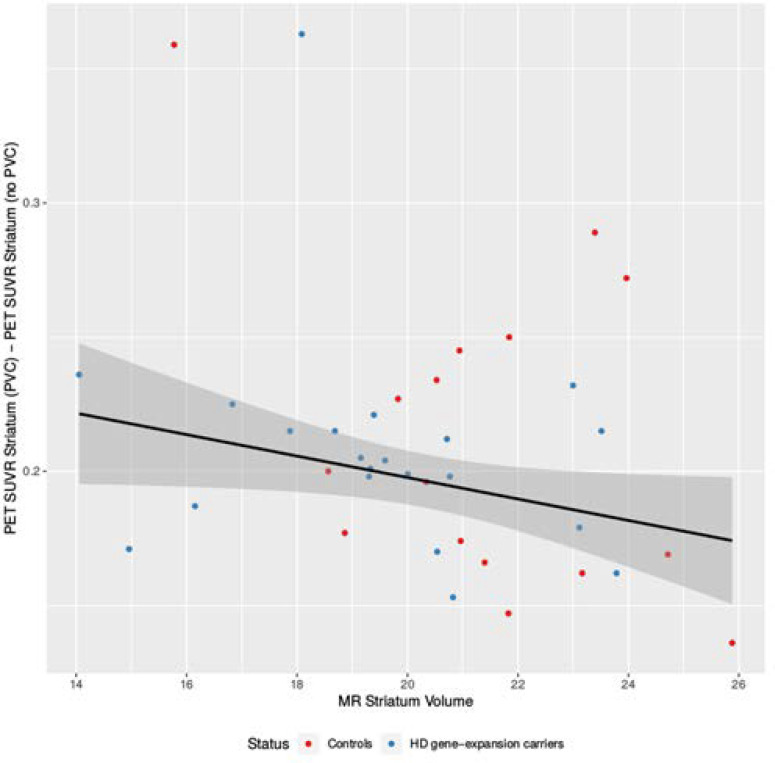
**Plot of the difference in striatal 2-[**^**18**^**F]FDG PET SUVR with and without partial volume correction vs. the absolute striatal volume (mL) of both HD gene-expansion carriers (blue dots) and controls (red dots).** The slope is -0.023, p = 0.01, one sided. The plot shows that the influence of partial volume correction is largest in objects with the smaller absolute MRI volumes. The variation of differences in striatal 2-[^18^F]FDG PET SUVR could signify the importance of other factors such as shape and location of the structure that may influence PVC performance. The four top values were eliminated as outliers and not included in the analysis.

Documenting the effect of PVC, **Fig 3** shows the linear regression of the difference in striatal 2-[^18^F]FDG PET SUVR with and without PVC vs. the absolute striatal volume (mL). Four values, that changed more than 0.25 SUVR, were eliminated as outliers and not included in the analysis. The elimination did not change the overall results(slope -0.025; p = 0.03 without vs slope -0.023; p = 0.01 with elimination).

The accuracy, sensitivity and specificity were calculated using ROC analyses on HDGECs (**Table 3)**. There was a significant positive association between striatal PVC 2-[^18^F]FDG PET SUVR and MRI volume, but not in other regions (**Table 4**).

**Table 3:** Diagnostic accuracy for partial volume corrected 2-[^18^F]FDG PET SUVR and intracranial volume corrected MRI volume of bilateral striatum for the separation of controls and Huntington’s disease gene-expansion carriers.

	Sensitivity	Specificity	Accuracy	Threshold
PVC 2-[^18^F]FDG PET SUVR	0.91	0.53	0.74	1.64
ICV MRI Volume	0.57	0.88	0.71	1.24%

Calculated thresholds of the average value of striatum from both hemispheres with related sensitivity, specificity and accuracy. SUVR: standardized uptake value relative to cerebellar cortex.

**Table 4:** Association between PVC 2-[^18^F]FDG PET SUVR and MRI volume, corrected for ICV.

	Hippocampus	Amygdala	Thalamus	Cortex	Striatum
Correlation	-0.05	-0.10	-0.15	0.09	0.44
P-value	0.76	0.55	0.36	0.61	0.01
Corrected p-value	0.76	0.76	0.76	0.76	0.03*

Association between metabolism and volume in the different structures, corrected for intracranial volume and multiple comparisons. Significant results after correction for multiple comparisons by Bonferroni-Holm marked by *. SUVR: standardized uptake value relative to cerebellar cortex.

The SPM analysis showed a single significant cluster encompassing both Putamina bridging across the third ventricle with significant correlation to the CAPs score (**Table 5**). The only significant peak surviving a conservative threshold of p <0.05, however, was located in the third ventricle, while the peaks located in the Putamina, was not significant on the voxel level, only at the cluster level (**S2 Fig** in S1 File).

**Table 5:** SPM analysis of the negative correlation of normalized 2-[^18^F]FDG uptake to CAPs score in premanifest HD gene-expansion carriers.

		Stereotactic coordinates				
L/R	Region	X	Y	Z	T-value	P_FWEcorr_
L	third ventricle	-8	-4	10	4.6	<0.05
R	Putamen	26	4	6	3.6	0.23
L	Putamen	-24	6	2	3.5	0.23

The cluster level significance was p <0.05 P_FWEcorr_ for clusters > 200 voxels at p <0.01 uncorrected.

P_FWEcorr_: Age adjusted voxel level significance corrected for multiple independent comparisons using Family-Wise- Error (FWE).

L: Left hemisphere.

R: Right hemisphere.

## 4. Discussion

In this study we examined a group of premanifest HDGECs, that represent a wide spectrum of premanifest HD, acquiring data simultaneously using a short scan session with 2-[^18^F]FDG PET on a hybrid PET/MRI scanner. Hybrid PET/MRI imaging has not earlier been applied in studies of the partial volume effect in HDGECs but may prove valuable in determining imaging outcome measures of interventions in presymptomatic HDGECs and probably also symptomatic HD. Visually we identified no HD related MRI findings. In evaluating the metabolism of the different brain structures, we chose the cerebellum as reference. A prior study have described early changes in this structure and suggests that the degeneration of cerebellum is early and independent from the striatal atrophy [27]. We found no visual atrophy of the cerebellum of our participants. If atrophy had been found, the use of the cerebellum as reference structure would only underestimate the relative degree of hypometabolism in the striatum. We divided the HDGECs into groups based on visual evaluation of their 2-[^18^F]FDG PET scan. We found that a HDGEC with a visually abnormal 2-[^18^F]FDG PET scan is likely to have motor symptoms within the next five years based on their CAP_S_ score, but if the scans were visually evaluated as normal, we could not predict their time to becoming motor manifest (**Fig 1** and **S1 Fig** in S1 File).

### 4.1 The relevance of the 2-[^18^F]FDG PET imaging technique in Huntington’s disease

Applying PVC, we found a statistically significant lower relative striatal metabolism in the premanifest HDGECs but not a significantly reduced striatal volume which could indicate that the hypometabolism evolve before the atrophy. It should be noted that the striatal volume was on average 8% lower in the HDGEC group, but this was not significant when corrected for the number of comparisons. The question of whether atrophy or hypometabolism comes first has been addressed in earlier studies as part of the diagnostic evaluation [6,11,28] but after the identification of the gene mutation, the aim of imaging studies have in general shifted towards identifying biomarkers of progression and time to disease onset [8,29,30]. As part of a longitudinal program, van Oostrom et al., in a cohort of 27 premanifest HDGECs, found normal striatal imaging in 88% for MRI, 67% in 2-[^18^F]FDG PET and in 50% using the selective Dopamine D2 receptor antagonist, [^11^C]-Raclopride and PET. This indicates that 2-[^18^F]FDG and [^11^C]Raclopride PET scanning, especially the latter, were more sensitive imaging modalities than MRI when detecting changes in relation to premanifest HD [31]. The importance of identifying biomarkers is even more relevant now considering the ongoing clinical trials on potential therapies [32], highlighting the need for an easily accessible biomarker that can register small and early changes. Measurement of the reduced glucose metabolism, have in a prospective 2-[^18^F]FDG PET study of 43 premanifest HDGECs by Ciarmiello et al. shown to improve the prediction of age at onset by 37% [33] which supports our results of 2-[^18^F]FDG PET being a more precise modality for registering early changes. The combined accuracy of metabolism and volume measures was not higher than the metabolism alone, because of the large overlap in the information of MRI and metabolism. In general, the accuracy is not very high, which is expected (**Fig 2)** because our participants are premanifest and relatively far from onset.

### 4.2 Correlation between striatal metabolism and volume in Huntington’s disease

Our study shows a significant association between striatal PVC 2-[^18^F]FDG PET SUVR and MRI volume corrected for ICV and no significant association between PVC 2-[^18^F]FDG PET SUVR and other cerebral regions of interest. The association is positive, meaning the larger the volume, the higher the metabolism, even when the confounding effects of volume on metabolism are considered. We found a significant negative correlation between both striatal metabolism and striatal volume and CAP_S_ score, indicating that the closer to expected motor onset, the smaller volume and lower metabolism which is also indicating that these measures could help us in the prediction of time to onset. Based on the determination coefficient, R^2^, we found that the measure of metabolism explained more of the variation in CAP_S_ than the MRI volume measures did. These results were corroborated by a voxel-based analysis, that did not find correlation in other areas with the exceptions of the third ventricle (**Table 5**, **S2 Fig** in S1 File). This is likely a spurious effect caused by white matter atrophy and ventricular enlargement (**Fig 1**), known to be present already in the presymptomatic stages [4]. It further underlines the necessity of PVC, as voxel based analysis may confound results by misrepresenting structural differences between groups, as physiological.

### 4.3 The importance of partial volume correction

A limitation to our study is, as in most other HD imaging studies, a limited number of participants and the cross-sectional design. Despite the limited number of participants, our study shows the importance of using PVC, especially in HD where the structures of interest are small. When looking into the correlation between metabolism and CAP_S_ score, we found a steeper slope when not correcting for PVE, -0.43 vs. -0.47, pushing an extra HDGEC under the -2SD, this HDGEC is marked with red in Fig 2. The negative slopes (**Fig 2)** along with the linear regression of the differences (**Fig 3)** shows that the influence of PVE is highest in structures with smaller volumes and that the effect creates a larger difference between the HDGECs with low and with high CAP_S_ scores. Not using PVC could therefore lead to underestimation of the metabolism in the affected regions. However, addressing the question of sequential order of the decrease in metabolism and atrophy further would imply a higher number of participants and a longitudinal study design using hybrid scans and applying PVC.

## Supporting information

S1 File (DOCX)
