# Supplementary material for: Hybrid 2-[18F] FDG PET/MRI in premanifest Huntington’s disease gene-expansion carriers: The significance of partial volume correction
Source: PLoS One. 2021 Jun 11;16(6):e0252683. doi: 10.1371/journal.pone.0252683 (PMC8195345; doi:10.1371/journal.pone.0252683)
Supplement: S1 File — (DOCX) [file pone.0252683.s001.docx]

**Supplementary material**

**The 5 standard clinical sequences of the 20 min lasting MRI protocol:**

1. A sagittal 3D T1-weighted magnetization-prepared rapid acquisition gradient-echo (MPRAGE) sequence (TR/TE/TI 1900/2.44/900 ms, flip angle 9˚, 192 slices, voxel size 1.0×1.0×1.0 mm3; acquisition time 5:04 min);

2. A T2-weigthed fluid-attenuated inversion-recovery (FLAIR) sequence (TR/TE/TI 9000/58/2500 ms, flip angle 130˚, 35 slices, voxel size 0.9×0.9×5.0 mm3, acquisition time 2:26 min);

3. A T2-weighted fast spin-echo (TSE) sequence (TR/TE 5000/83 ms, flip angle 150˚, 35 slices, voxel size 0.9×0.9×5.0 mm3, acquisition time of 1:27 min,

4. A susceptibility weighted image (SWI) sequence (TR/TE 25/20 ms, flip angle 15˚, 112 slices, voxel size 1.1×1.0×1.0 mm3, acquisition time of 5:53 min;

5. A diffusion weighted image (DWI) sequence (TR/TE 4200/94 ms, 26 slices, voxel size 1.9×1.9×5.0 mm3, acquisition time of 3:53 min.

| **Table S1. Supplementary table** |
| --- |
| **Exclusion criteria** |
| Claustrophobia |
| Pacemaker or neurostimulator |
| Pregnancy or breastfeeding |
| Exposure to radiation within the last year |
| Metallic Prostheses in the inner ear |
| Ongoing alcohol or drug abuse |
| Other native language than Danish |
| Mechanical heart valve |

**Figure S1: Plot of CAP_S_ scores vs. visual grading of disease severity in** 2-[^18^F]FDG **PET images**

**
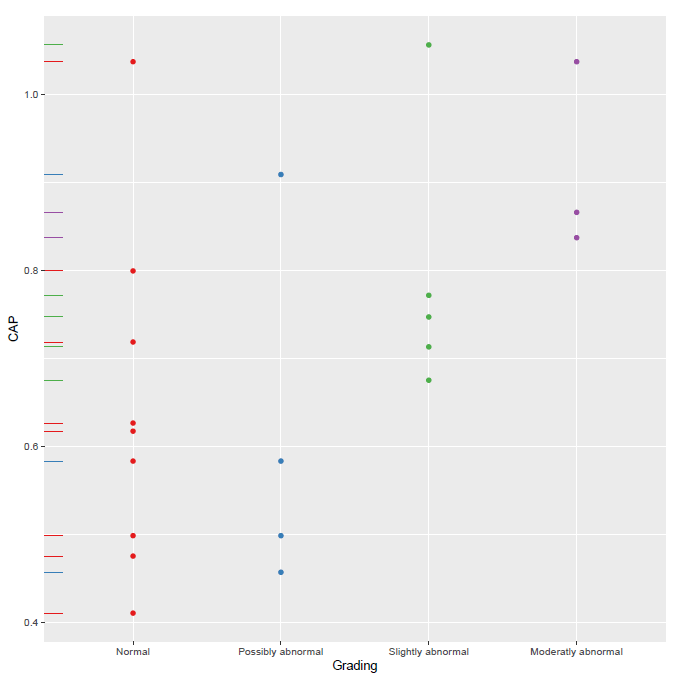
**

**Figure S1.** Plot of CAP_S_ scores from all HD gene-expansion carriers categorized by their visual score of disease severity on 2-[^18^F]FDG PET. There is an overall significant correlation between CAP_S_ and disease severity on 2-[^18^F]FDG PET, but a subgroup of HD gene-carriers with high CAP_S_ scores were not manifesting on 2-[^18^F]FDG PET, which illustrates the variation in CAP_S_ scores of the 2-[^18^F]FDG PET scans evaluated as normal.

**Figure S2: The negative correlation of** 2-[^18^F]FDG **uptake with CAPs score**

**Figure S2.** Overlay of areas where normalized 2-[^18^F]FDG uptake (SUVR) correlate negatively to the CAPs score in premanifest HD gene-expansion carriers projected onto an average anatomical MRI template in MNI SPM space. The cross-hair points to the right Putamen. The bridging activity connecting the two Putamina is likely to be a spurious correlation brought on by the increasing size of the third ventricle with CAPs score. The image has a threshold at p < 0.01 uncorrected for clusters > 200 voxels. The cluster level significance was p <0.05 P_FWE corr_.
